# Characterizing the Retinal Phenotype of the Thy1-h[A30P]α-syn Mouse Model of Parkinson’s Disease

**DOI:** 10.3389/fnins.2021.726476

**Published:** 2021-09-07

**Authors:** Lien Veys, Joyce Devroye, Evy Lefevere, Lien Cools, Marjan Vandenabeele, Lies De Groef

**Affiliations:** ^1^Research Group of Neural Circuit Development and Regeneration, Department of Biology, KU Leuven, Leuven, Belgium; ^2^Department of Biomedical Sciences, Leuven Brain Institute, Leuven, Belgium

**Keywords:** retina, visual system, alpha-synuclein, transgenic mouse model, Parkinson’s disease

## Abstract

Despite decades of research, disease-modifying treatments of Parkinson’s disease (PD), the second most common neurodegenerative disease worldwide, remain out of reach. One of the reasons for this treatment gap is the incomplete understanding of how misfolded alpha-synuclein (α-syn) contributes to PD pathology. The retina, as an integral part of the central nervous system, recapitulates the PD disease processes that are typically seen in the brain, and retinal manifestations have emerged as prodromal symptoms of the disease. The timeline of PD manifestations in the visual system, however, is not fully elucidated and the underlying mechanisms are obscure. This highlights the need for new studies investigating retinal pathology, in order to propel its use as PD biomarker, and to develop validated research models to investigate PD pathogenesis. The present study pioneers in characterizing the retina of the Thy1-h[A30P]α-syn PD transgenic mouse model. We demonstrate widespread α-syn accumulation in the inner retina of these mice, of which a proportion is phosphorylated yet not aggregated. This α-syn expression coincides with inner retinal atrophy due to postsynaptic degeneration. We also reveal abnormal retinal electrophysiological responses. Absence of selective loss of melanopsin retinal ganglion cells or dopaminergic amacrine cells and inflammation indicates that the retinal manifestations in these transgenic mice diverge from their brain phenotype, and questions the specific cellular or molecular alterations that underlie retinal pathology in this PD mouse model. Nevertheless, the observed α-syn accumulation, synapse loss and functional deficits suggest that the Thy1-h[A30P]α-syn retina mimics some of the features of prodromal PD, and thus may provide a window to monitor and study the preclinical/prodromal stages of PD, PD-associated retinal disease processes, as well as aid in retinal biomarker discovery and validation.

## Introduction

Despite decades of research, disease-modifying treatments of Parkinson’s disease (PD), the second most common neurodegenerative disease worldwide, remain out of reach (Guo et al., 2018; Veys et al., 2019). It has been suggested that one of the principal reasons for this treatment gap is the lack of accurate and timely diagnosis. Traditionally, diagnosis is based on the cardinal motor symptoms of PD (tremor, rigidity, bradykinesia, and postural instability), which only arise years after a long non-symptomatic phase during which a large proportion of the dopaminergic cells in the substantia nigra are lost (Jankovic, 2008). In order to preserve brain function, therapies -and hence diagnosis- should be focused on the preclinical (asymptomatic) and prodromal (early symptomatic) stages (Forsaa et al., 2010; Mahlknecht et al., 2015; Hustad and Aasly, 2020). In 2017, new diagnostic criteria for PD have been defined by the International Parkinson Disease and Movement Disorders Society (Postuma and Berg, 2017; Marsili et al., 2018), whereby the probability of an individual to develop PD is now calculated based on several predictors, such as age, environmental predictors, prodromal signs, genetic risk variables, and biomarker testing (Postuma et al., 2016). Constant updating of these diagnostic criteria is required as more insights into early stage PD emerge (Postuma and Berg, 2017).

The retina has become a target organ in the search for early biomarkers, relevant diagnostic criteria and techniques that are amenable to population-wide patient screening and disease monitoring. As an integral part of the central nervous system (CNS), the eye can be considered a window to the brain. The visual pathway has shown to be an excellent model system to gain insight into classical neurodegenerative diseases, as both retina and brain are often affected by these diseases and share disease processes (e.g., neurodegeneration, inflammation, aggregation of misfolded proteins, mitochondrial dysfunction; Armstrong, 2009; Martínez-Lapiscina et al., 2014; Rahimi et al., 2015; Veys et al., 2019; Kashani et al., 2021). Therefore, it is not surprising that in many PD patients, one or more visual symptoms are described, such as decreased visual acuity, spatial contrast sensitivity, and color vision (Price et al., 1992; Archibald et al., 2011; Armstrong, 2011; Bodis-Wollner, 2013; Guo et al., 2018). Retinal dysfunction at least partially contributes to these deficits (Bertrand et al., 2012; Mazzarella and Cole, 2016). This is corroborated by retinal imaging via optical coherence tomography (OCT) and with electroretinography (ERG) measurements, which revealed, respectively, retinal nerve fiber layer (NFL), ganglion cell layer (GCL), inner plexiform layer (IPL), and inner nuclear layer (INL) thinning (Shrier et al., 2012; Adam et al., 2013; London et al., 2013; Spund et al., 2013; Lee et al., 2014; Bodis-Wollner et al., 2014b; Boeke et al., 2016; Aydin et al., 2018; Matlach et al., 2018); and abnormalities of the photopic b-wave, scotopic oscillatory potentials (OPs), and P50 component of the pattern ERG in PD patients (Nightingale et al., 1986; Gottlob et al., 1987; Burguera et al., 1990; Ikeda et al., 1994; Peppe et al., 1992, 1995, 1998; Langheinrich et al., 2000; Sartucci et al., 2006; Garcia-Martin et al., 2014; Nowacka et al., 2015; Kashani et al., 2021). Histopathological studies have revealed pathological manifestations that may underlie these changes in *in vivo* measures, including a reduction in dopamine levels in the retina (Nguyen-Legros, 1988; Harnois and Di Paolo, 1990; Chorostecki et al., 2015), reduced density and complexity of dopaminergic neurons (Ortuño-Lizarán et al., 2020) and melanopsin-positive retinal ganglion cells (RGCs; Ortuno-Lizaran et al., 2018b), and, finally, the presence of alpha-synuclein (α-syn) and phosphorylated (S129) α-syn (p-α-syn) inclusions in the retina (Beach et al., 2014; Ho et al., 2014; Bodis-Wollner et al., 2014a; Ortuno-Lizaran et al., 2018a; Veys et al., 2019). Importantly, p-α-syn deposits in the retina accumulate in parallel with the brain, already during the prodromal stage of PD, and are associated with PD severity (Ortuno-Lizaran et al., 2018a). This reinforces that retinal biomarkers have a high potential for PD diagnosis and disease monitoring.

Further research into the (temporal) relationship between retinal biomarker alterations and neurodegenerative changes in the brain is needed, however, for retinal biomarkers to be adopted in the clinic. Longitudinal and prospective studies in PD patients and patients at risk of developing PD will be essential to assess the value of retinal biomarkers for PD (Kashani et al., 2021). Animal models of PD, on the other hand, can support these studies, by providing a framework in which the correlation between retinal biomarkers and disease manifestations can be explored and novel insights into the molecular and cellular changes underlying the retinal manifestations of PD can be obtained (Santano et al., 2011; Normando et al., 2016; Price et al., 2016; Mammadova et al., 2018, 2021; Veys et al., 2019). Altogether, the wide availability of technologies for non-invasive high-resolution ocular imaging, such as OCT, is a clear advantage over current brain imaging techniques (De Groef and Cordeiro, 2018) and, collectively, visual function measures, ERG, and retinal imaging could offer a multimodal biomarker approach for PD diagnosis, stratification, and monitoring (Guo et al., 2018; Turcano et al., 2018; Veys et al., 2019).

In this study, we aim to fill the need for well-characterized preclinical models to study retinal alternations in PD. We characterized the retinal phenotype of the Thy1-h[A30P]α-syn mouse model, by studying α-syn accumulation, neurodegeneration, inflammation, synaptic integrity, and retinal function. The brain phenotype of this mouse model has been studied before, yet the retinal phenotype remains untouched (Kahle et al., 2000; Neumann et al., 2002; Freichel et al., 2007; Ekmark-Lewen et al., 2018). Here, we used *in vivo* retinal imaging and electrophysiology measurements with high clinical translatability, combined with *post mortem* histological studies, to map the timeline of retinal disease manifestations in these mice.

## Materials and Methods

### Animals

Thy1-h[A30P]α-syn mice (C57BL/6 background, RRID:MGI:2652214) and corresponding wild type (WT) controls, were bred under standard laboratory conditions (Kahle et al., 2000). Both female and male mice were used at 4, 8, 12, 15, and 18 months of age. All experiments were performed according to the European directive 2010/63/EU and in compliance with protocols approved by the KU Leuven institutional ethical committee.

### (Immuno)histochemistry

Prior to eye dissection, mice were euthanized by an intraperitoneal injection of 60 mg/kg sodium pentobarbital (Dolethal, Vetoquinol) followed by transcardial perfusion with saline and 4% paraformaldehyde (PFA). Next, eyes were either fixed in 1% PFA for 4 h at 4°C and embedded in paraffin, or in 4% PFA for 1 h at RT for wholemount preparations. The latter were post-fixed for 1 h in 4% PFA for another hour.

Seven-micrometer sagittal paraffin sections were deparaffinized and stained with hematoxylin (Sigma) and eosin (Sigma) and mounted with Distyrene Plasticizer Xylene mounting medium (Sigma). For Thioflavin S histological staining, sections were stained for 5 min with Thioflavin S (Sigma, 1/200 in 1:1 distilled water and ethanol) and mounted with mowiol (Sigma). For immunohistochemistry, sections were incubated overnight with one or two of the following primary antibodies: human specific α-syn (1/5000; Millipore, clone Syn211 [36-008] RRID:AB_310817), α-syn (1/1000; produced and kindly provided by V. Baekelandt, KU Leuven, for double staining with p-α-syn), p-α-syn (1/5000; Elan Pharmaceuticals), p62 (1/200; Proteintech [#55274-1-AP], RRID:AB_11182278), Brn3a (1/750; Santa Cruz Biotechnology, c-20 [#sc-31984], RRID:AB_2167511), tyrosine hydroxylase (TH; 1/1000; Millipore [#AB152], RRID:AB_390204), choline acetyltransferase (ChAT; 1/100; Millipore [#AB144P], RRID:AB_2079751), VGLUT1 (1/1000, Synaptic Systems [#135 302], RRID:AB_887877), Prox1 (1/500; Biolegend [PCB-238C]), Homer1 (1/500; Synaptic Systems [#160 003], RRID:AB_887730), glial fibrillary acidic protein (GFAP; 1/1000; Dako [#Z0334], RRID:AB_10013382), or aquaporin 4 (AQP4; 1/10000; Alomone labs [AQP-004], RRID:AB_2039734). For α-syn, Brn3a, TH, ChAT, Prox1, and Homer1, antigen retrieval with heated citrate buffer (20 min, 95°C) was used, while no antigen retrieval treatment was used for VGLUT1 and proteinase K (5 min, 20 μg/ml, Qiagen) antigen retrieval was used for GFAP stainings. Fluorescent labeling was performed using an Alexa-488 labeled secondary antibody (Invitrogen) for Brn3a, TH, ChAT, Prox1, VGLUT1, and GFAP staining, or with a fluorescein or cyanine 3 tyramid signal amplification kit (PerkinElmer) for p-α-syn, α-syn, and Homer1 stainings. Finally, slides were counterstained with 4′,6-diamidino-2-phenylindole and mounted with mowiol.

For wholemount immunohistochemistry, tissue permeabilization was achieved by a freeze-thaw step (15 min, −80°C), followed by overnight incubation with one of the following primary antibodies: p-α-syn (1/5000; Elan Pharmaceuticals), TH (1/1000; Millipore [#AB152], RRID:AB_390204), melanopsin (1/5000; Advanced Targeting Systems [#AB-N38], RRID:AB_1608077), or ionized calcium-binding adapter molecule 1 (Iba-1; 1/1000; Wako [#019-19741], RRID:AB_839504). Subsequently, fluorescent labeling was performed using an Alexa-488 labeled secondary antibody (Invitrogen) and wholemounts were mounted with mowiol.

### Image Analysis

Imaging was performed using a FV1000 confocal or FV1000-M multiphoton microscope (Olympus) or a conventional epifluorescence microscope (DM6, Leica).

Image analyses were performed with Fiji software (Schindelin et al., 2012). For retinal wholemounts, the entire perimeter of the wholemount was outlined and its area measured prior to analysis. For sections, five sections per mouse were investigated, including the central section containing the optic nerve head, and the sections located 210 and 420 μm anterior/posterior. On each section, analysis was done over a distance of 300 μm at four locations per section. For α-syn, TH and GFAP, the immunopositive area was measured in the inner retina (from the retinal NFL until the INL included), while for AQP4 both the outer retina (from OPL to ONL) and inner retina were measured and for VGLUT1 and Homer1, only the IPL was included (Van Hove et al., 2020). For cell counting, both on wholemounts and sections, Fiji “Cell Counter” plugin was used. Microglia density and morphology were quantified as described in Davis et al. (2017) on projection images of z-stack (step size 1.5 μm) pictures of Iba-1 stained wholemounts (Davis et al., 2017).

### Optical Coherence Tomography

Optical coherence tomography imaging was performed as described before (Sergeys et al., 2019; Vandenabeele et al., 2021). Briefly, after pupil dilatation with tropicamide (0.5%, Tropicol, Théa), the retina of anesthetized animals was imaged (1000 A-scans, 100 B-scans, 1.4 × 1.4 mm, Bioptigen Envisu R2200). Retinal layer thickness was measured using InVivoVue Diver (v 3.0.8, Bioptigen) software, at 16 locations in the central retina spaced around the optic nerve head, and averaged per mouse.

### Electroretinography

Electroretinography was performed as described before (Sergeys et al., 2019; Vandenabeele et al., 2021). Full-field flash dark-adapted electroretinograms were measured at increasing flash intensities of 0.003, 0.01, 0.1, 1, 2.5, and 7.5 cd^∗^s/m^2^. Electroretinograms were analyzed using Espion software (v6.59.9, Diagnosys), as shown in Supplementary Figure 1. To analyze the OPs on the rising part of the b-wave, a band pass filter (75–300 Hz) was used. The positive scotopic threshold response (pSTR) was measured at 1 × 10^–4^ cd^∗^s/m^2^. 1 week after baseline ERG or pSTR measurement, mice were intraperitoneally injected with benserazide hydrochloride (12.5 g/kg, Sigma) and L-DOPA (25 g/kg, Sigma) 50′ and 30′ prior to ERG/pSTR measurement, respectively.

### Statistical Analysis

Statistical analyses were performed using GraphPad Prism (v8.4.3, GraphPad, RRID:SCR_002798). The number of animals (*n*) used is depicted on the figures and the statistical analyses are indicated in the figure legends. Data are presented as mean ± SEM. Differences were considered statistically significant for two-sided *p*-values < 0.05 (^∗^*p* < 0.05; ^∗∗^*p* < 0.01; ^∗∗∗^*p* < 0.001; and ^****^p < 0.0001).

## Results

### Retinal Accumulation of (Phosphorylated) α-syn in Thy1-h[A30P]α-syn Mice

**FIGURE 1:**
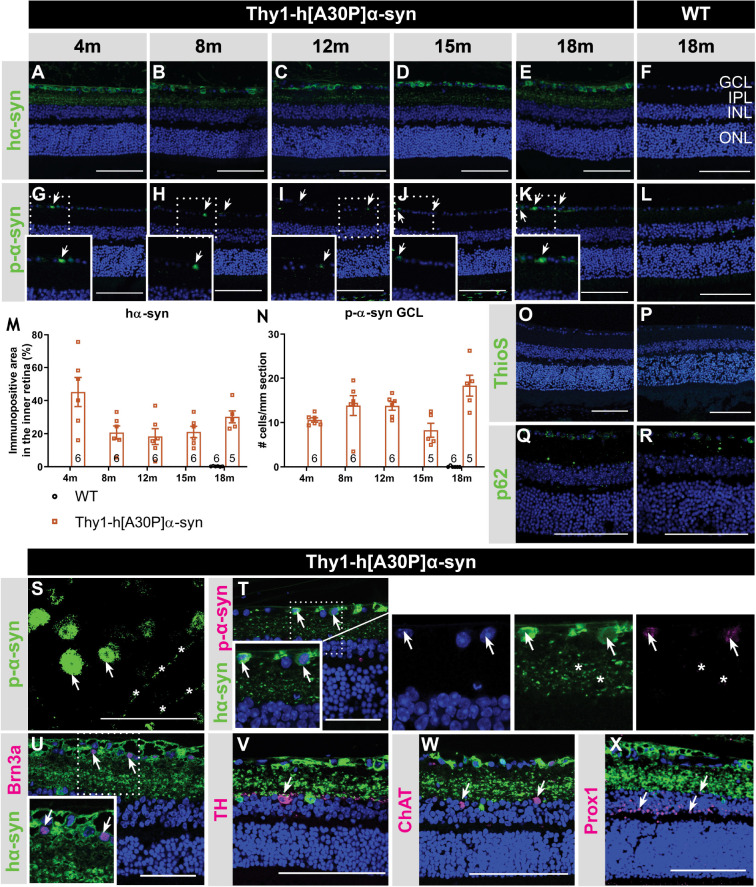
Inner retinal hα-syn expression is accompanied by α-syn phosphorylation, yet no ThioS positive aggregation or p62 accumulation, in the retina of Thy1-h[A30P]α-syn mice. Representative images of hα-syn immunostainings **(A–E)**; p-α-syn immunostainings **(G–K)**; and ThioS staining **(O)** on retinal sections of α-syn mice at 4, 8, 12, 15 and 18 months of age. **(F,L,P)** No staining was observed in the WT controls, at any age (only 18 months shown here). **(M,N)** Quantitative analysis of the hα-syn fluorescent area and counting of the p-α-syn positive cells did not reveal an increase of hα-syn expression in the inner retina or p-α-syn cell density in α-syn mice with age. **(O,P)** No ThioS positive inclusions were found in the retina of transgenic nor wild type animals in any of the age groups. **(Q,R)** No difference in retinal p62 accumulation or localization was detected between transgenic and wild type animals at 18 months of age. **(S)** p-α-syn immunostaining on a retinal wholemount of an α-syn mouse showed p-α-syn localization in cell bodies (arrows) and neurites (asterisks). **(T)** Double staining of hα-syn with p-α-syn revealed clear colocalization. **(U–X)** Double staining of hα-syn with Brn3a, TH, ChAT and Prox1 revealed expression of Brn3a in hα-syn positive cells, yet no colocalization in dopaminergic and cholinergic cells. Scale bar: 100 μm **(A–R, V–X)** or 50 μm **(S–U)**; GCL, ganglion cell layer; INL, inner nuclear layer; IPL, inner plexiform layer; and ONL, outer nuclear layer.

α-syn expression, phosphorylation, and aggregation was studied in the retina of WT and Thy1-h[A30P]α-syn mice (α-syn mice) of various ages, using (immuno)stainings for transgenic human α-syn, phosphorylated (serine-129) α-syn (p-α-syn; detecting both human and rodent α-syn), thioflavin S (ThioS) and p62. Conform with previously published data of Veys et al. (2019), hα-syn expression was observed in neuronal cell bodies in the GCL, in neurites in the retinal NFL and IPL and in dispersed cell bodies in the INL of 4-, 8-, 12-, 15-, and 18-month-old Thy1-h[A30P]α-syn mice (Figures 1A–F,T; Veys et al., 2019). The hα-syn positive cell types in the inner retina comprise RGCs, as shown by double staining with Brn3a (Figure 1U), and amacrine cells, based on their morphology and location (Figures 1V–X). Furthermore, the accumulation of hα-syn in dopaminergic, (nor)adrenergic, cholinergic, or AII amacrine cells was ruled out based on the lack of colocalization with TH, ChAT, and Prox1 positive cells, respectively (Figures 1V–X; Müller et al., 2017). Quantitative analysis of the hα-syn fluorescent area did not reveal any progressive changes in hα-syn expression in the inner retina of α-syn mice with aging (Figure 1M). Next, a fraction of α-syn was phosphorylated, most prominently in cell bodies and neurites in the GCL (Figures 1G–L,S,T), and this did not change with age (Figure 1N), not even in end-stage diseased animals with severe signs of hind limb paralysis (data not shown). At 18 months of age, only 34 ± 8% of strongly α-syn positive cells in the GCL also contained p-α-syn. Finally, we assessed p62 and ThioS labeling to investigate α-syn ubiquitination and aggregation, respectively. At 18 months of age, no p62 accumulation nor relocalization were observed in the retina of α-syn mice as compared to WT mice (Figures 1Q,R), and no ThioS positive aggregates were found in the retina of transgenic nor WT animals (Figures 1O,P). Of note, although no accumulation of ThioS-positive or p62-positive cellular inclusions was detted in the Thy1-h[A30P]α-syn PD mouse model, we cannot exclude that oligomeric, prefibrillar, or non-fibril α-syn conformers contribute to the retinal phenotype observed in these mice (Lashuel et al., 2013; Roberts and Brown, 2015; Cascella et al., 2021). This needs to be explored in follow-up studies.

Altogether, these data show that, while both α-syn overexpression and phosphorylation are present in the retina of Thy1-h[A30P]α-syn mice already at a young age, α-syn aggregation and ubiquitination do not manifest.

### Synaptic Degeneration in the Retina of Old Thy1-h[A30P]α-syn Mice

**FIGURE 2:**
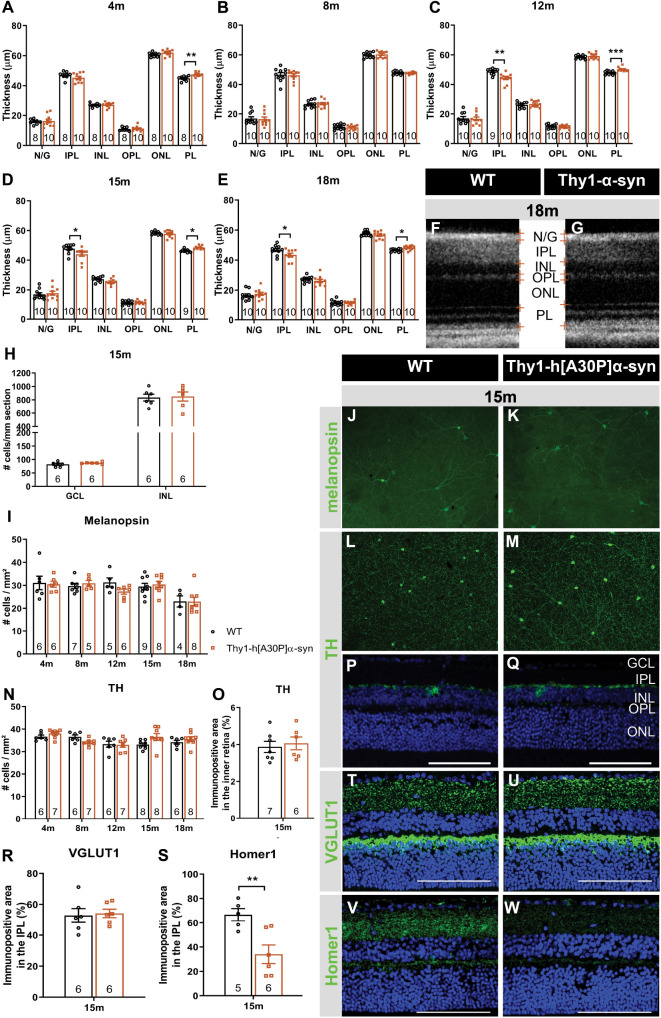
Outer retinal thickening and inner retinal thinning, associated with loss of postsynaptic labeling, in Thy1-h[A30P]α-syn mice. **(A–E)** Longitudinal OCT measurements in 4- **(A)**, 8- **(B)**, 12- **(C)**, 15- **(D)**, and 18-month-old **(E–G)** mice, revealed significant differences in retinal layer thickness between α-syn and WT mice of 4 months (PL thickening), 15 months (PL thickening and IPL thinning), and 12 and 18 months of age (PL thickening and IPL thinning). **(H)** Cell counts on hematoxylin and eosin-stained sections in the GCL and in the INL did not reveal significant differences between transgenic animals and WT controls at 15 months of age. **(I–W)** Representative images of retinal wholemounts stained for melanopsin **(J,K)** and TH **(L,M)**, and of retinal sections stained for TH **(P,Q)**, VGLUT1 **(T,U)**, and Homer-1 **(V,W)**, of 15-month-old α-syn and WT mice. Counting the number of melanopsin- **(I)** and TH- **(N)** positive cells on retinal wholemounts revealed no significant differences between transgenic and WT animals. No significant differences were uncovered in TH plexus **(O)** and VGLUT1 **(R)** immunopositive area, yet a strong decrease of the Homer1 **(S)** signal was seen. Scale bar: 100 μm; Two-Way ANOVA with Tukey multiple comparisons *post hoc* test **(I–N)**. Unpaired *t*-test (per retinal layer; **A–F,O,R,S**): *p < 0.05; **p < 0.01; and ***p < 0.001. N/G, retinal nerve fiber layer + GCL; GCL, ganglion cell layer; INL, inner nuclear layer; IPL, inner plexiform layer; ONL, outer nuclear layer; OPL, outer plexiform layer; and PL, photoreceptor layer.

Spectral domain OCT was applied in a longitudinal *in vivo* experiment to measure the thickness of the retinal layers in α-syn mice and WT controls, early in their life (4 and 8 months) and at 12, 15, and 18 months of age (Figures 2A,B,D–F). At 4 months of age, a minor yet significant thickening of the photoreceptor layer (PL) was found in the α-syn mice (*p* = 0.0023; Figure 2A). This difference in retinal thickness persisted at 12, 15, and 18 months (*p* = 0.0009, *p* = 0.0130, and *p* = 0.0122; Figures 2C–G). Furthermore, at 12 months, α-syn mice also displayed thinning of the IPL, which persisted at 15 and 18 months (*p* = 0.0034 at 12 months, *p* = 0.0336 at 15 months, *p* = 0.0444 at 18 months; Figures 2C–G).

As retinal thinning is typically a sign of neurodegeneration, we next performed a more in-depth analysis of different subpopulations of inner retinal neurons at 15 months of age to clarify the origin of the observed IPL thinning. Given that the IPL consists of neurites emerging from cell bodies in both the GCL and INL, cell density was assessed in these layers on hematoxylin and eosin-stained sections. No overt neurodegeneration was seen in α-syn mice (Figure 2H). Additionally, a detailed analysis of disease-relevant neuronal subtypes, also at 15 months of age, revealed that cell numbers of intrinsically photosensitive RGCs (melanopsin positive) in the GCL and of dopaminergic (TH positive) amacrine cells in the INL (Figures 2I–N) were not affected. However, IPL thinning may also occur due to dendrite or synapse loss, a pathological process that is known to precede loss of neuronal cell bodies. In line with the preservation of dopaminergic cell bodies (cfr. above), we found that the dopaminergic plexus of the retina, measured as the TH-immunopositive area in the inner retina, was unaltered in α-syn mice of 15 months of age (Figures 2O–Q). However, taking a closer look at the synaptic integrity of the IPL, via immunostainings with the established pre- and postsynaptic markers VGLUT1 and Homer1, we revealed loss of postsynaptic contacts yet preservation of the presynaptic terminals in 15-month-old transgenic mice (Figures 2R–W). Altogether, these findings suggest that synaptic degeneration in the retina underlies the observed IPL thinning.

### Electrophysiological Changes in the Retina of Thy1-h[A30P]α-syn Mice With Aging

**FIGURE 3:**
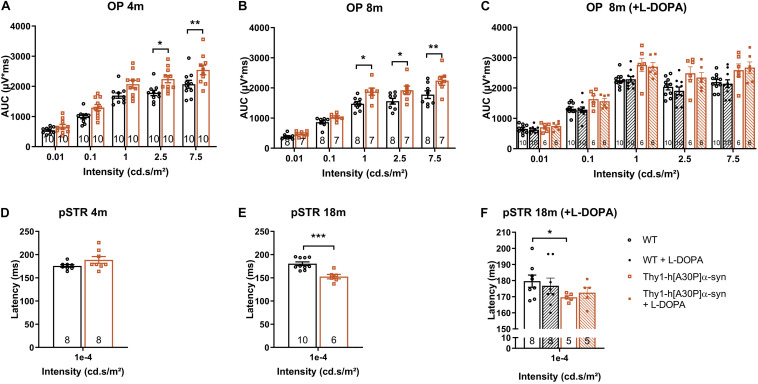
Electrophysiological changes in the retina of older Thy1-h[A30P]α-syn mice cannot be alleviated by L-DOPA treatment. ERG was used to measure the electrophysiological responses of different retinal cell types. **(A,B)** Quantification of the OPs, measured as the area under the curve (AUC), revealed larger OPs in 4- and 8-month-old α-syn mice as compared to WT controls for light stimuli with high intensity. **(D,E)** Quantification of pSTR response did not reveal any differences at 4 months of age, yet a shorter pSTR latency time was observed in 18-month-old transgenic mice as compared to WTs. **(C,F)** L-DOPA treatment did not have an overt rescue effect on observed OP **(C)** and pSTR **(F)** differences in α-syn mice. Repeated measures Two-Way ANOVA **(A–C)** with Bonferroni’s multiple comparisons *post hoc* test or unpaired *t*-test **(D–F)**: *p < 0.05; **p < 0.01; and ***p < 0.001. Full ERG data is shown in Supplementary Figure 1.

In a next series of experiments, we sought to further identify the neuronal cell types that are affected in the α-syn mouse and to establish whether neuronal dysfunction can be detected already at younger ages compared to the OCT thinning that only become apparent at 12 months. Indeed, neuronal death is often preceded by functional changes, and these prodromal manifestations of the disease are of particular interest for biomarker development (Nowacka et al., 2015; Barber et al., 2017; Turcano et al., 2018; Hustad and Aasly, 2020). First, OPs as a read-out for amacrine cell function, were assessed. Already at 4 months, the area under the curve was larger in α-syn mice as compared to WT animals for high intensity light stimuli (2.5 cd^∗^s/m^2^: *p* = 0.0137; 7.5 cd^∗^s/m^2^: *p* = 0.0094), and this effect persisted in older transgenic animals of 8 (1 cd^∗^s/m^2^: *p* = 0.0191; 2.5 cd^∗^s/m^2^: *p* = 0.0452; 7.5 cd^∗^s/m^2^: *p* = 0.0050), 12 (1 cd^∗^s/m^2^: *p* = 0.0034; 2.5 cd^∗^s/m^2^: *p* = 0.0039; 7.5 cd^∗^s/m^2^: *p* = 0.0023), and 18 months of age (1 cd^∗^s/m^2^: *p* = 0.0001; Figures 3A,B and Supplementary Figure 1F). Second, we measured RGC function via the pSTR. Not yet at 4 months, but at 8, 12, and 18 months, the pSTR latency time was shorter in α-syn mice as compared to WT controls (*p* = 0.0082 at 8 months, *p* = 0.0119 at 12 months, and *p* = 0.0006 at 18 months; Figures 3D–F and Supplementary Figure 1G). *a*- and *b*-wave measurements were unaltered, indicating normal functioning of the photoreceptors, bipolar cells, and Müller glia (Supplementary Figures 1B–E).

In PD patients, visual defects have been attributed to malfunctioning of the dopaminergic retinal neurons -which constitute a subtype of amacrine cells-, which is supported by the fact that ERG abnormalities can be alleviated by L-DOPA treatment (Ikeda et al., 1994; Djamgoz et al., 1997; Peppe et al., 1998; Turcano et al., 2018). Hence, we assessed the effect of systemic L-DOPA treatment 30 min prior to the ERG measurement in a second, independent study. We found that L-DOPA did not fully reverse the effects of genotype on the OPs in 8-month-old mice, nor the pSTR latency in 18-month-old mice (Figures 3C,F). These findings are in line with the absence of dopaminergic degeneration as observed in the immunohistological studies (*cfr*. above). Overall, ERG changes in the α-syn mice suggest that amacrine cells and RGCs become dysfunctional with age, yet TH immunostainings showed that it is unlikely that a selective loss of dopaminergic neurons underlies this phenotype.

### No Signs of Neuroinflammation in the Retina of Thy1-h[A30P]α-syn Mice

**FIGURE 4:**
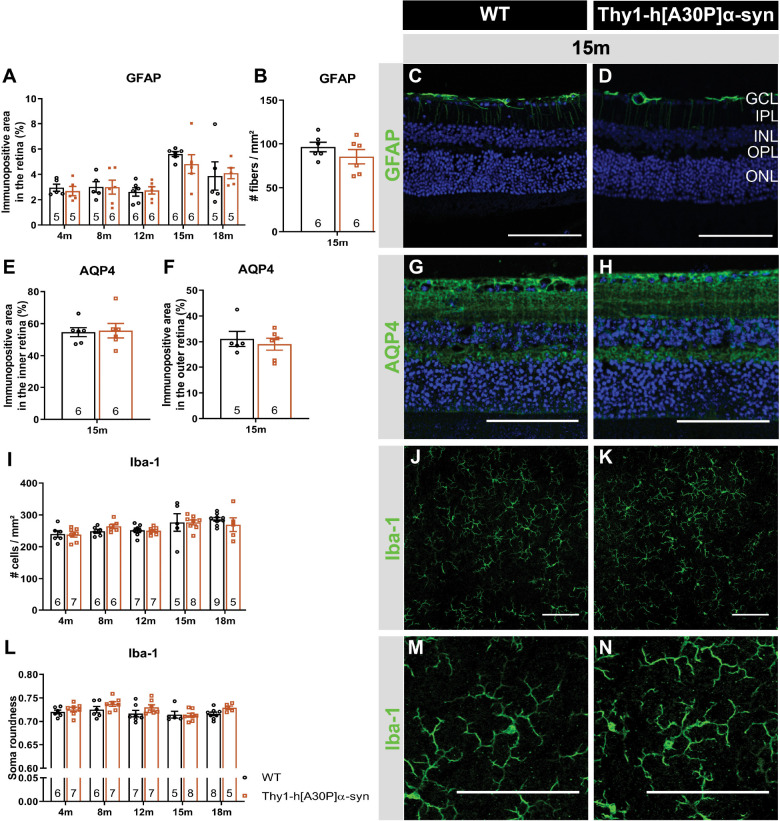
Macroglia and microglia reactivity and water homeostasis appear normal in Thy1-h[A30P]α-syn mice. Representative images of retinal cross-sections stained for GFAP **(C,D)** and wholemounts stained for Iba-1 **(J,K,M,N)** and cross-sections stained for AQP4 **(G,H)** in 15-month-old α-syn and WT mice. **(A,B)** When measuring the GFAP immunopositive area and the number of radial fibers in the inner retina, no differences in macroglia reactivity were uncovered between transgenic and WT animals in any of the age groups. **(I,L)** No differences in Iba-1^+^ cell density and cell soma roundness, indicative of microgliosis, were observed. **(E,F)** AQP4 immunopositive area or localization in the inner versus outer retina of α-syn mice versus age-matched WT animals was similar. Two-Way ANOVA with Sidak’s multiple comparisons *post hoc* test **(A,I,L)** or unpaired *t*-test **(B,E,F)**. Scale bar: 100 μm.

Previous studies demonstrated that α-syn triggers neuroinflammation, and that, in turn, inflammation increases α-syn phosphorylation and pathology in synucleinopathies (Lee et al., 2010; Tansey and Goldberg, 2010; Ramirez et al., 2017; Ferreira and Romero-Ramos, 2018). Furthermore, retinal inflammation has been linked to both swelling of the outer retina and ERG deviations, and may therefore underlie -at least in part- the OCT and ERG abnormalities that we observed in the Thy1-h[A30P]α-syn mice (Mirza and Jampol, 2013; Petzold, 2016; Pisa et al., 2021; Xia et al., 2021). Hence, we next investigated macroglia and microglia reactivity and water homeostasis in the retina. First, Müller glia and astrocytes were investigated. Analysis of GFAP immunostainings on retinal cross-sections of α-syn versus WT mice did not reveal differences in immunofluorescent area at 4, 8, 12, 15, and 18 months of age and radial fiber density at 15 months of age between the two genotypes, although an expected aging effect was present (Figures 4A–D). Second, the cause of outer retinal swelling was further investigated by measuring the expression of AQP4 (Figures 4G,H). AQP4 is a water channel expressed by the Müller glia, of which differences in expression levels and cellular localization have been linked to retinal edema and neuroinflammation (Amann et al., 2016). In AD patients, it was found to be overexpressed in the brain and associated with blood-brain barrier disruption (Foglio and Luigi Fabrizio, 2010; Fukuda and Badaut, 2012). However, no genotypic difference in immunofluorescent area nor localization in the inner versus outer retinal layers was revealed in mice of 15 months old (Figures 4E,F). Third, microgliosis was investigated on retinal wholemounts stained for Iba-1 (Figures 4J,K,M,N). Cell density did not differ in transgenic versus WT mice at any of the selected ages (Figure 4I). Furthermore, we investigated cell morphology, to probe for changes in soma roundness as a sign of microglia reactivity (Davis et al., 2017). However, no difference in cell body roundness of Iba-1^+^ cells was observed between the two genotypes (Figure 4L). In conclusion, this data suggests that retinal inflammation nor edema underlie the OCT and ERG abnormalities that we observed in the α-syn mice.

## Discussion

In recent years, neurodegenerative disease research is increasingly focusing on the pre- and early symptomatic stages of disease, when the cascade of neurodegenerative events has only just started and a sufficiently large pool of neurons still remains that can be rescued with disease-modifying treatments to preserve brain function. To identify and take opportunity of this early time window for treatment, however, novel biomarkers and inexpensive, minimally invasive, and widely available screening and diagnostic tests are needed. These may be found in the retina. As an integral part of the CNS, the retina recapitulates many of the PD-related neurodegenerative process in the brain. Indeed, a multitude of OCT and ERG studies has shown that neuronal dysfunction and degeneration affects the retina of PD patients (Garcia-Martin et al., 2014; Boeke et al., 2016; Aydin et al., 2018; Veys et al., 2019). Furthermore, accumulating evidence of retinal dopamine deficits and α-syn misfolding suggest that this is the result of the same disease processes that also drive neurodegeneration in the brain (Guo et al., 2018; Ortuno-Lizaran et al., 2018a; Veys et al., 2019; Ortuño-Lizarán et al., 2020). It remains to be explored, however, what the correlation between the PD manifestations in the brain and retina is, and whether the mechanisms behind these manifestations are the same. A deeper understanding of this will be essential for the rational use of retinal biomarkers for PD diagnosis, monitoring and/or stratification, and will also aid research into novel retinal biomarkers. Animal research will remain an essential complement to the extensive clinical studies that are obviously needed, offering flexibility in study subjects and read-outs to dig into the cellular and molecular changes that characterize the PD retina and dictate the retinal biomarker results. Up till now, multiple studies have investigated the brain phenotype of PD animal models, yet retinal manifestations have received little attention (Santano et al., 2011; Normando et al., 2016; Price et al., 2016; Veys et al., 2019). Mammadova et al. investigated the retinal phenotype of the TgM83 mouse model. This transgenic mouse is characterized by α-syn accumulation mainly in the outer retina and p-α-syn pathology in both outer and inner retina, and thereby only partially mimics the inner retina pathology seen in PD patients (Mammadova et al., 2018, 2021). In addition, and in contrast to the Thy1-h[A30P]α-syn model, neuroinflammation, and photoreceptor cell loss were seen in the TgM83 model, again partially reflecting human disease – where also microglia reactivity was seen (Tansey and Goldberg, 2010; Ferreira and Romero-Ramos, 2018; Mammadova et al., 2018). Both in the Thy1-h[A30P]α-syn and TgM83 mice, and in contrast to reports on the human PD retina (Archibald et al., 2009; Mammadova et al., 2018; Ortuño-Lizarán et al., 2020), TH immunoreactivity was unaltered (Table 1). The lack of dopaminergic degeneration, even in end-stage animals (data not shown), highlights the limitations of the available transgenic mouse models in recapitulating the full complexity of human disease. Of note, this is in line with findings in the brain, where a lack of progressive neurodegeneration has been reported for several rodent PD models (Lim and Ng, 2009; Dawson et al., 2010; Kin et al., 2019). Furthermore, the diverging retinal manifestations observed in these two mouse models might result from the use of distinct promoters (Thy1 versus Prp) and/or different mutated forms of α-syn (A30P versus A53T), which might influence the aggregation process (Flagmeier et al., 2016). By examining the retina of the Thy1-h[A30P]α-syn PD mouse model, we aim to establish a research model with a retinal α-syn expression pattern that more closely resembles α-synucleinopathy in PD patients. We believe that such as model is valuable to investigate the retina-brain connection in PD and thereby propel retinal biomarker discovery and validation research and fundamental studies of the role of α-syn in health and disease.

**TABLE 1:** Summary of the phenotypical alterations observed in the retina of PD patients, Thy1-h[A30P]α-syn mice.

**PD patients**	**References**	**Thy1-h[A30P]α -syn mice**	**TgM83 mice**
α-syn in GCL, IPL, and INL	Beach et al., 2014; Ho et al., 2014; Bodis-Wollner et al., 2014a	α-syn in GCL, IPL, and INL	α-syn in ONL and INL
p-α-syn positive cell bodies and neurites in GCL	Beach et al., 2014; Ortuno-Lizaran et al., 2018a	p-α-syn positive cell bodies and neurites in GCL	p-α-syn labeling in outer and inner retina
			p-Tau (Thr231) in OPL and GCL
Thinning of NFL, GCL, IPL, and INL (OCT)	Shrier et al., 2012; Adam et al., 2013; Spund et al., 2013; Lee et al., 2014; Bodis-Wollner et al., 2014b; Matlach et al., 2018	Thinning of IPL (OCT)	Thinning of ONL (histology)
		Thickening of PL (OCT)	
Decreased TH levels and TH-positive cell density in INL Decreased TH + plexus complexity	Nguyen-Legros, 1988; Harnois and Di Paolo, 1990; Chorostecki et al., 2015; Ortuño-Lizarán et al., 2020	Preserved TH-positive cell density in INL	Preserved TH levels in INL
		Preserved TH + plexus size in IPL	
Decreased melanopsin-positive cell density in GCL and dendritic tree complexity	Ortuno-Lizaran et al., 2018b	Preserved melanopsin-positive cell density in GCL	
Increased microglial reactivity (Iba-1)	Doorn et al., 2014; Ferreira and Romero-Ramos, 2018	No microglial reactivity (Iba-1)	Increased microglial reactivity (CD11b, CD68)
No macroglial reactivity (GFAP)	Mirza et al., 1999	No macroglial reactivity (GFAP)	Macroglial reactivity (GFAP)
RGC, bipolar and amacrine cell dysfunction (ERG):	Nightingale et al., 1986; Gottlob et al., 1987; Burguera et al., 1990; Ikeda et al., 1994; Peppe et al., 1992, 1995, 1998; Langheinrich et al., 2000; Sartucci et al., 2006; Garcia-Martin et al., 2014; Nowacka et al., 2015; Kashani et al., 2021	RGC and amacrine cell dysfunction (ERG):	
− diminished responses of the photopic b-wave, scotopic oscillatory potentials and P50 component of the pattern ERG reversed by L-DOPA		− supernormal responses of the scotopic oscillatory potentials and pSTR (starting at 4 and 8 months, respectively)	
− reversed by L-DOPA		− no overt response to L-DOPA	

*Retinal manifestations on the TgM83 mouse model were described in Mammadova et al. (2018). OCT, optical coherence tomography; NFL, nerve fiber layer; GCL, ganglion cell layer; IPL, inner plexiform layer; INL, inner nuclear layer; ONL, outer nuclear layer; OPL, outer plexiform layer; PL, photoreceptor layer; ERG, electroretinography; OPs, oscillatory potentials; pSTR, positive scotopic threshold response; and TH: tyrosine hydroxylase.*

We revealed that, from a young age onward, α-syn overexpression can be observed in the inner retina of α-syn mice, alongside a fraction of phosphorylated α-syn in RGC neurites and somata; an observation that complies with previously described (p)-α-syn localization in the retina of PD patients (Table 1; Ortuno-Lizaran et al., 2018a; Veys et al., 2019). Despite the lack of ThioS positive protein aggregates and accumulation of the Lewy body marker p62, α-syn overexpression did result in thinning of the inner retina in α-syn mice from the age of 12 months, similar to the inner retinal remodeling seen in PD patients (Table 1; Shrier et al., 2012; Adam et al., 2013; Spund et al., 2013; Lee et al., 2014; Bodis-Wollner et al., 2014b). Our data revealed that neurodegeneration of dopaminergic amacrine cells or melanopsin positive RGCs cannot account for this IPL thinning uncovered with OCT imaging. Instead, synapse loss may underlie this retinal atrophy. Indeed, significant changes in the density of Homer1+ postsynaptic -yet not VGLUT1+ presynaptic- terminals in the IPL underscore the OCT alterations. Postsynaptic terminals in the IPL come from RGCs and amacrine cells, neurons for which we also observed hα-syn overexpression and abnormal ERG responses (Connaughton, 1995). Furthermore, synapse loss has been shown to occur early in the neurodegenerative process, for example in the retina of glaucoma models and patients, or in the brain of AD or PD models and patients (Selkoe, 2002; Della Santina et al., 2013; Purro et al., 2014; Bellucci et al., 2016; Subramanian and Tremblay, 2021). More specifically, a decrease in synaptic volume in of pre- and post-synapses has been reported in the striatum of PD patients (Bellucci et al., 2016; Reeve et al., 2018; Gcwensa et al., 2021). Of note, an age-related decrease of postsynaptic retinal proteins was also observed in the plexiform layers of *Octodon degus*, the only rodent with naturally occurring AD (Chang et al., 2020).

The retinal atrophy and synapse loss observed in α-syn mice is accompanied by functional alterations, which were uncovered using ERG. These were striking for several reasons. First, amacrine cell responses were supernormal in α-syn mice. Although abnormal OPs are also typically seen in PD patients, these ERG alterations tend to decrease rather than increase in human patients (Table 1; Gottlob et al., 1987; Burguera et al., 1990; Ikeda et al., 1994; Nowacka et al., 2015). Remarkably, these supernormal ERG responses in α-syn mice coincide with a thickening of the PL, which might be caused by local edema or swelling of the photoreceptors (Devos et al., 2005; Archibald et al., 2009). Interestingly, this outer retinal swelling was also seen in the early disease stages of a rotenone-induced PD rat model, where it was suggested to be linked to increased mitochondrial biogenesis in the highly energy demanding photoreceptor cells (Normando et al., 2016). Outer retinal thickening has also been observed to co-occur with supernormal ERG measurements in the retina of the 3×Tg-AD Alzheimer’s (Chiquita et al., 2019a). Furthermore, both supernormal scotopic ERG measurements and PL layer thickening have been related to a mild inflammatory phenotype in the early stages of retinal pathology linked to multiple sclerosis (Mirza and Jampol, 2013; Petzold, 2016). Yet, with the measurements used in this study, no abnormalities in AQP4 water channels and no inflammatory response of the macro- and microglia was detected. Second, an equally striking observation in this study is the increased conduction velocity of RGC electrophysiological responses in older animals, reminiscent of the RGC hyperactivity in early AD disease stages of 5×FAD mice (Araya et al., 2021). In AD models, amyloid-beta overproduction can lead to neuronal network hyperexcitability (Kazim et al., 2021). As AD and PD are both neurodegenerative proteinopathies and amyloid-beta and α-syn biology show many parallels, one could hypothesize that similar neuronal network hyperexcitability events might occur in PD too (Goedert, 2015). This hypothesis is supported by our data on synaptic integrity, which show preservation of presynaptic integrity yet loss of postsynaptic density. The postsynaptic density Homer1 proteins link metabotropic glutamate receptors to intracellular effectors, mediating the glutamate inducible effects in postsynaptic RGCs and amacrine cells (Connaughton, 1995). Dysregulation of extracellular glutamate concentrations at the synapse can lead to excess release of glutamate, which is known to induce hyperexcitability in postsynaptic neurons (Gasparini and Griffiths, 2013). An alternative explanation for the supernormal ERG responses by RGCs might relate to the physiological role of α-syn at the synapse, where it is suggested to associate with synaptic vessels and to influence neurotransmitter release (Sulzer and Edwards, 2019). Since α-syn overexpression inhibits synaptic vesicle exocytosis, one could hypothesize that decreased exocytosis might disturb the tightly maintained balance that is involved in synaptic regulation (Sulzer and Edwards, 2019). Finally, the electrophysiological alterations observed in this study were, in contrast to ERG changes in PD patients, not reversed by L-DOPA treatment (Table 1; Archibald et al., 2009). Along with the observed lack of dopaminergic cell loss in the retina and the absence of hα-syn in dopaminergic amacrine cells in the α-syn mice, this suggests a dopamine-independent mechanism underlying the ERG alterations. Which neuronal subtype(s) account for the observed electrophysiological abnormalities should be elucidated in future research via more advanced electrophysiology studies, e.g., using patch clamping or microelectrode arrays (Obien et al., 2015; Chiquita et al., 2019b).

Besides generating insights into the (patho)physiological role of α-syn and the disease processes that lead to the retinal PD phenotype, we postulate that the α-syn mouse may also aid the understanding of the retina-brain connection. Indeed, the α-syn mouse is characterized by hα-syn overexpression in neuronal cell bodies and neurites in the brain and spinal cord (Table 2; Kahle et al., 2000; Freichel et al., 2007) and p-α-syn and oligomeric α-syn were detected in brainstem, midbrain, and hippocampus of 8-month-old transgenic mice. In addition, older mice also develop proteinase K-resistant α-syn deposits, ubiquitin-positive neuritic and cell body inclusions, and ThioS reactive α-syn species in various CNS regions (Table 2; Neumann et al., 2002; Schell et al., 2009). This synucleinopathy in the brain is accompanied by astrogliosis and dopaminergic neurodegeneration (Ekmark-Lewen et al., 2018), and led to a variety of behavioral changes in fine motor performance, learning, and memory, finally leading to paralysis and premature death around the age of 18 months (Table 2; Freichel et al., 2007; Ekmark-Lewen et al., 2018). We conclude that the rather subtle retinal phenotype stands in marked contrast to findings in the brain of these mice, exposing the organotypic heterogeneity of the retina compared to other brain structures. Notably, this heterogeneity may be exploited as a strength in future research, and aid the understanding of disease mechanisms and selective vulnerability in different locations in the CNS.

**TABLE 2:** Overview of the reported phenotypical alterations in the brain and spinal cord of Thy1-h[A30P]α-syn mice, in relation to observations in the retina.

**Observations in the brain and spinal cord**	**Time point of first observation**	**References**	**Own observations in the retina**	**Time point of first observation**
**Functional read-outs**				
Decreased fine motor performance (beam transversal test)	2 months, worsens with age	Ekmark-Lewen et al., 2018		
Lower general activity and more risk-taking (multivariate concentric square field test)	8 months	Ekmark-Lewen et al., 2018		
Impaired spatial learning and memory (Morris water maze)	12 months	Freichel et al., 2007		
Impaired fear conditioning (freezing behavior after foot shock)	12 months	Freichel et al., 2007		
Higher locomotor activity	12 months	Freichel et al., 2007		
Impaired motor behavior (rotarod test)	17 months	Freichel et al., 2007		
(Hind limb) paralysis	18 months	Freichel et al., 2007		
Premature death	18 months	Freichel et al., 2007		
Decreased frequency of spontaneous excitatory postsynaptic currents (electrophysiology)	1 month	Chesselet et al., 2012	OP alterations (ERG)	4 months
			pSTR latency alterations (ERG)	18 months
**Histopathology**				
α-syn overexpression in neuronal cell bodies and neurites in the brain and spinal cord	6 month	Kahle et al., 2000	α-syn overexpression in neuronal cell bodies and neurites in the inner retina	4 months
p-α-syn positive neurons in spinal cord and brainstem	1 months	Freichel et al., 2007	p-α-syn positive neurons in GCL	4 months
Oligomeric α-syn in brainstem, midbrain and hippocampus	8 months	Ekmark-Lewen et al., 2018		
PK-resistant α-syn in brain	9 months	Neumann et al., 2002; Freichel et al., 2007		
Ubiquitin-positive inclusions in pontine reticular nuclei and ventral horn of the spinal cord	12 months	Neumann et al., 2002		
ThioS reactive species in brainstem	16 months	Schell et al., 2009	No ThioS reactivity detected	
Decreased TH immunoreactivity in central midbrain regions	8 months	Ekmark-Lewen et al., 2018	No changes in TH immunoreactivity	
Increased GFAP immunoreactivity in brainstem	8 months	Neumann et al., 2002; Ekmark-Lewen et al., 2018	No changes in GFAP immunoreactivity	
Limited inflammatory response (increase in Mac2^+^ immune cells)	8 months	Ekmark-Lewen et al., 2018	No changes in Iba-1 immunoreactivity	
No reports of neurodegeneration			PL thickening (OCT)	4 months
			IPL thinning (OCT)	15 months

*OCT, optical coherence tomography; PL, photoreceptor layer; IPL, inner plexiform layer; INL, inner nuclear layer; ERG, electroretinography; OPs, oscillatory potentials; pSTR, positive scotopic threshold response; TH, tyrosine hydroxylase; PK, proteinase K; and ThioS, thioflavin S.*

Irrespective of the differences in the retina versus brain phenotype of the α-syn mice, this study highlights the potential of the retina for *in vivo* imaging and electrophysiology measurements with non-invasive techniques, such as OCT and ERG. Especially OCT, which detected retinal thinning in the inner retina in our transgenic mice similar to what has been described in the human PD retina, has the potential to become a low-cost, non-invasive tool for diagnosis and follow-up of PD disease progression (Shrier et al., 2012; Adam et al., 2013; Spund et al., 2013; Lee et al., 2014; Bodis-Wollner et al., 2014b). Importantly, these techniques have the advantage of being suitable for both patient and preclinical research, thereby providing relevant endpoint measures and enhancing the translatability of this research to the clinic.

In conclusion, this study uncovered morphological and electrophysiological abnormalities in the α-syn mouse retina. While this mouse model does not display dopaminergic neurodegeneration or neuroinflammation, its retina is characterized by a decreased density of postsynaptic terminals that may reflect neurotransmitter dysregulation and as such is linked to the observed ERG changes and IPL thinning. These pathological changes resemble the loss of synapses and neuronal dysfunction that are typically observed during the earliest stages of neurodegenerative diseases and are in line with a multitude of OCT and ERG studies in PD patients and animal models. The methodologies and the α-syn mouse model used in this study thus constitute a toolbox for research of the early, preclinical/prodromal stages of PD, and may aid fundamental research of PD-associated retinal disease processes, such as α-syn mediated synaptic dysfunction, as well as retinal biomarker discovery and validation.

## Data Availability Statement

The raw data supporting the conclusions of this article will be made available by the authors, without undue reservation.

## Ethics Statement

The animal study was reviewed and approved by KU Leuven institutional ethical committee.

## Author Contributions

LV and LD contributed to the conception of the study, elaborated on the study design, and wrote the manuscript. LV, JD, EL, LC, and MV performed the experimental work. LC edited the manuscript. All authors have read and approved the manuscript.

## Conflict of Interest

The authors declare that the research was conducted in the absence of any commercial or financial relationships that could be construed as a potential conflict of interest.

## Publisher’s Note

All claims expressed in this article are solely those of the authors and do not necessarily represent those of their affiliated organizations, or those of the publisher, the editors and the reviewers. Any product that may be evaluated in this article, or claim that may be made by its manufacturer, is not guaranteed or endorsed by the publisher.

## Abbreviations

α-syn: Alpha-synuclein
α-syn mice: Thy1-h[A30P] α-syn mice
AQP4: Aquaporin 4
ChAT: Choline acetyltransferase
CNS: Central nervous system
ERG: Electroretinography
DAPI: 4′,6-diamidino-2-phenylindole
GCL: Ganglion cell layer
GFAP: Glial fibrillary acidic protein
Iba-1: Ionized calcium-binding adapter molecule 1
INL: Inner nuclear layer
IPL: Inner plexiform layer
NFL: Nerve fiber layer
OP: Oscillatory potential
p-α-syn: Phosphorylated serine-129 α-syn
pSTR: Positive scotopic threshold response
RGC: Retinal ganglion cell
TH: Tyrosine hydroxylase
ThioS: Thioflavin S
WT: Wild type.
