# Supplementary material for: Characterizing the Retinal Phenotype of the Thy1-h[A30P]α-syn Mouse Model of Parkinson’s Disease
Source: Front Neurosci. 2021 Sep 7;15:726476. doi: 10.3389/fnins.2021.726476 (PMC8452874; doi:10.3389/fnins.2021.726476)
Supplement: Supplementary file 1 [file Data_Sheet_1.docx]

Supplementary Material

## Supplementary Figures

**
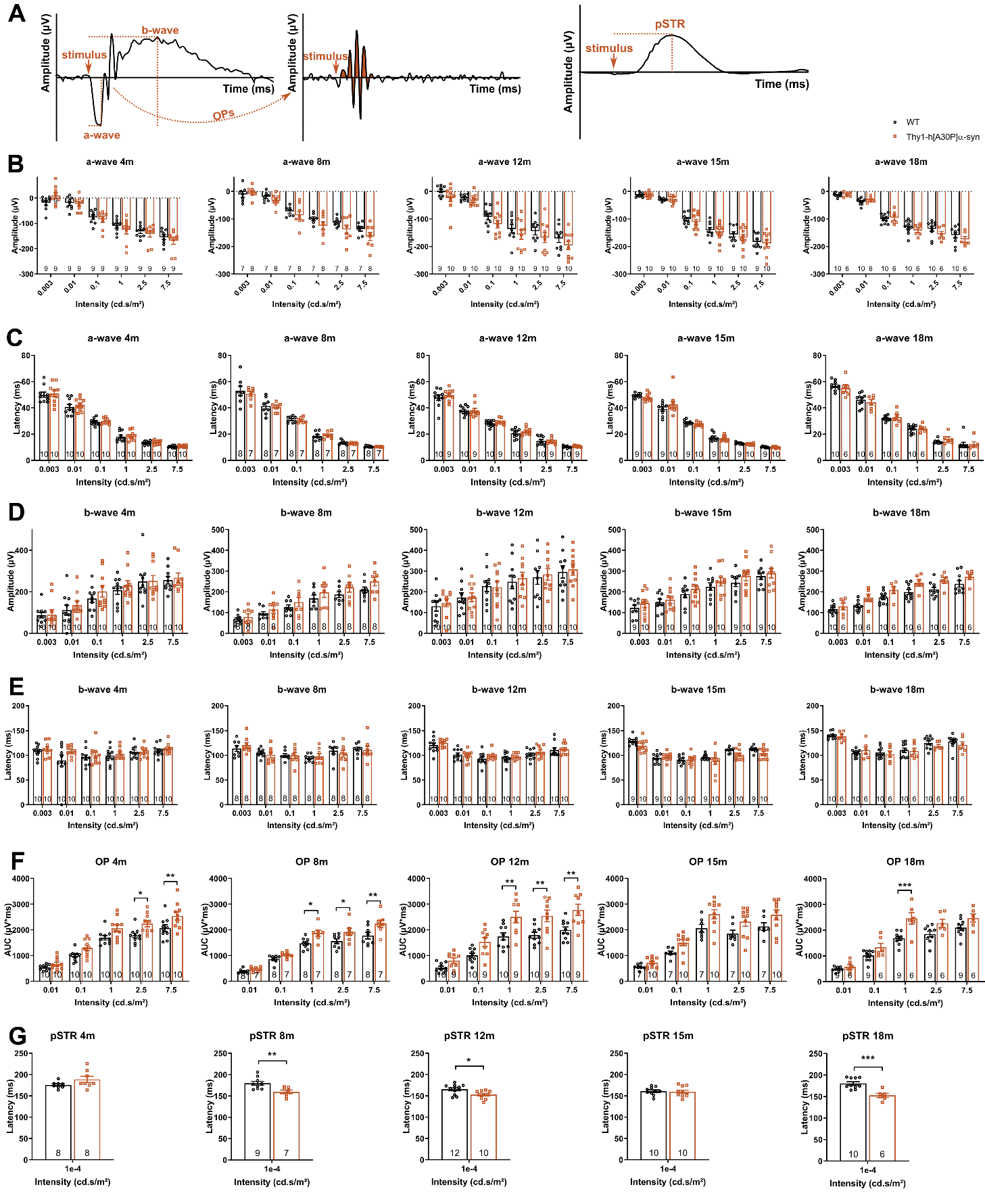
**

**Supplementary Figure 1.** **Electroretinograms show normal a- and b-waves yet abnormal OPs and pSTR responses in aging Thy1-h[A30P]α-syn mice.** **(A)** ERG was used to measure the electrophysiological responses of the different retinal cell types to light stimuli. A typical response pattern and parameters analysed, of both dark-adapted ERG and pSTR tests, are shown. **(B-G)** Quantification of the a-wave amplitude (B) and latency (C), the b-wave amplitude (D) and latency (E), the OP area (F) and the pSTR latency (G) of 4-, 8-, 12-, 15- and 18-month-old α-syn mice as compared to WT controls. Repeated measures Two-Way ANOVA with Bonferroni’s multiple comparisons post-hoc test (B-F) or unpaired t-test (G).
